# Automated estimation of parasitaemia of *Plasmodium yoelii*-infected mice by digital image analysis of Giemsa-stained thin blood smears

**DOI:** 10.1186/1475-2875-9-348

**Published:** 2010-12-01

**Authors:** Charles Ma, Paul Harrison, Lina Wang, Ross L Coppel

**Affiliations:** 1 Department of Microbiology, Monash University, Clayton Vic 3800, Australia; 2 Victoria Bioinformatics Consortium, Monash University, Clayton Vic 3800, Australia

## Abstract

**Background:**

Parasitaemia, the percentage of infected erythrocytes, is used to measure progress of experimental *Plasmodium *infection in infected hosts. The most widely used technique for parasitaemia determination is manual microscopic enumeration of Giemsa-stained blood films. This process is onerous, time consuming and relies on the expertise of the experimenter giving rise to person-to-person variability. Here the development of image-analysis software, named Plasmodium AutoCount, which can automatically generate parasitaemia values from *Plasmodium*-infected blood smears, is reported.

**Methods:**

Giemsa-stained blood smear images were captured with a camera attached to a microscope and analysed using a programme written in the Python programming language. The programme design involved foreground detection, cell and infection detection, and spurious hit filtering. A number of parameters were adjusted by a calibration process using a set of representative images. Another programme, Counting Aid, written in Visual Basic, was developed to aid manual counting when the quality of blood smear preparation is too poor for use with the automated programme.

**Results:**

This programme has been validated for use in estimation of parasitemia in mouse infection by *Plasmodium yoelii *and used to monitor parasitaemia on a daily basis for an entire challenge infection. The parasitaemia values determined by Plasmodium AutoCount were shown to be highly correlated with the results obtained by manual counting, and the discrepancy between automated and manual counting results were comparable to those found among manual counts of different experimenters.

**Conclusions:**

Plasmodium AutoCount has proven to be a useful tool for rapid and accurate determination of parasitaemia from infected mouse blood. For greater accuracy when smear quality is poor, Plasmodium AutoCount, can be used in conjunction with Counting Aid.

## Background

Infection of mice with rodent *Plasmodium *species is routinely conducted to evaluate the efficacy of drugs and vaccines against malaria. Parasitaemia, the percentage of infected erythrocytes, is used to monitor the progress of infection and recovery of infected mice. To date, the most widely used technique for parasitaemia determination in mouse blood is manual microscopic enumeration of Giemsa-stained blood films. This process is onerous, time consuming and relies on the expertise of the experimenters with consequent person-to-person variability [1]. An alternative method reported in recent years uses flow cytometry of fixed and stained cells. Although success has been reported [2-4], this approach has not been widely applied due to its limited specificity and the reliance on cytometry equipment, which is expensive and not commonly available in developing countries.

Image processing is an approach to automated determination of parasitaemia and uses more commonly available equipment:- a microscope with camera and a personal computer [1]. A number of studies have explored the possibility of software for automated parasitaemia counting and success has been reported for examining blood smears from *in vitro *culture [5-9]. However, software of this type, such as MalariaCount [8], cannot be applied to examination of blood smears from in *vivo *studies due to the presence of nucleated cells (e.g. lymphocytes) and other formed elements (platelets), as well as the increased number of reticulocytes in the blood of infected animals. Here the development of an image-analysis programme, Plasmodium AutoCount, which can automatically generate parasitaemia values from *Plasmodium*-infected mouse blood smears is reported. This programme has been used to measure daily parasitaemia in infected mice for an entire challenge infection and achieved results comparable to manual counting.

## Methods

### Giemsa-stained blood smears

Groups of mice were infected with *Plasmodium yoelii*-parasitized red blood cells after immunization with PyMSP1_19_, a well-characterized vaccine candidate, or with a saline control as described previously [10]. Daily from day 3 post-infection, one drop of blood was taken from the tail tip of each mouse and used to make a thin blood smear. The smears were fixed with 100% methanol for 2 min, stained with 10% Giemsa stain in Sorensen's buffer for 5 min, and air-dried.

### Image acquisition and standardization

An Olympus BX51 microscope with DP70 digital camera system was used to capture images of the smears. The smears were examined under oil immersion with a 100× objective and the numerical aperture set to 1.35. Automated exposure of fixed light intensity through a fully opened iris with one push white balance was used (although the image processing algorithm is robust to changes in background colour). Images were captured at a resolution of 1360×1024 pixels using the DP Controller programme and saved as TIFF files with the DP Manager programme.

### Manual counting tool

**Figure 1:**
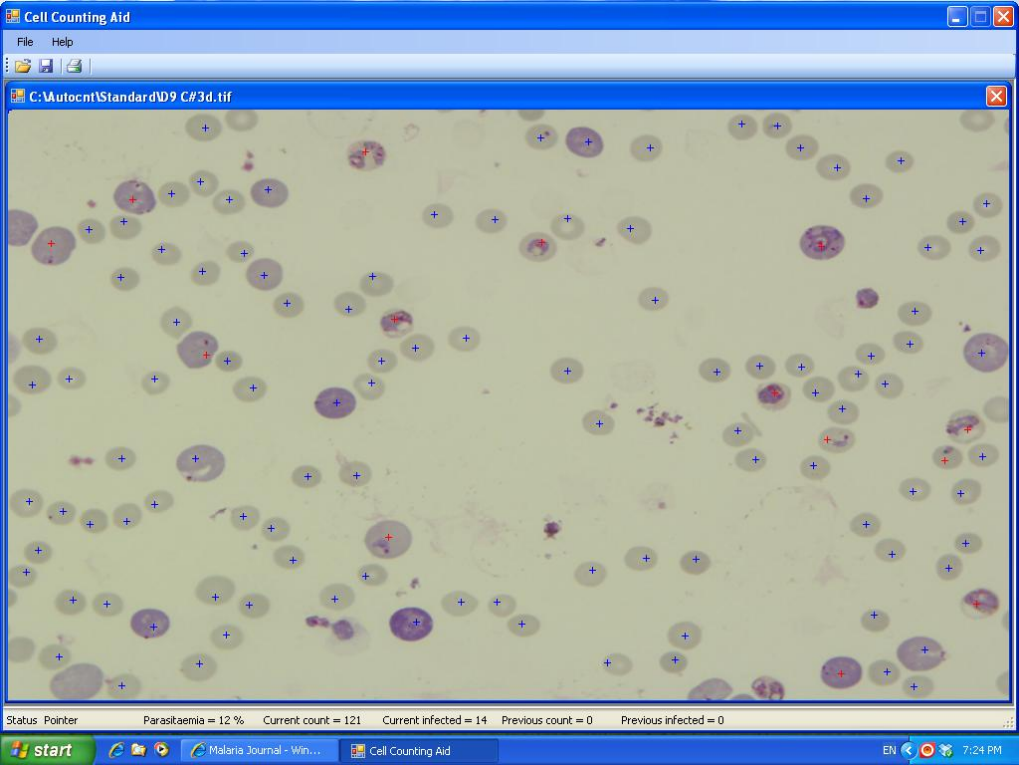
**Main screen of the semi-automatic counting programme "Cell Counting Aid"**. User points to each cell and click the appropriate button to mark it as infected or uninfected. The programme calculates parasitaemia value from the total number of cells and infected cells after each click.

A manual counting tool, Cell Counting Aid [11] that keeps records of counting outcome was developed. The information recorded includes the location of each cell within the image and the user's decision as to whether it is infected. A typical output screen of Cell Counting Aid is shown in Figure 1. This served as reference for the development of Plasmodium Autocount. It can also be used in its own right when the images of the smears are of poor quality. Cell Counting Aid runs on the Microsoft Windows platform and was written in Visual Basic™. The programme is a free software released under the General Public License (GPL) version 2 license. After an image is opened with the software, the operator uses the mouse to point to each cell and clicks the left button if the cell is uninfected or the right button if it is infected. Parasitaemia values are recalculated after each mouse click. The total number of cells and the total number of infected cells are recorded and can be exported to Excel™ for analysis.

### Programme design

**Figure 2:**
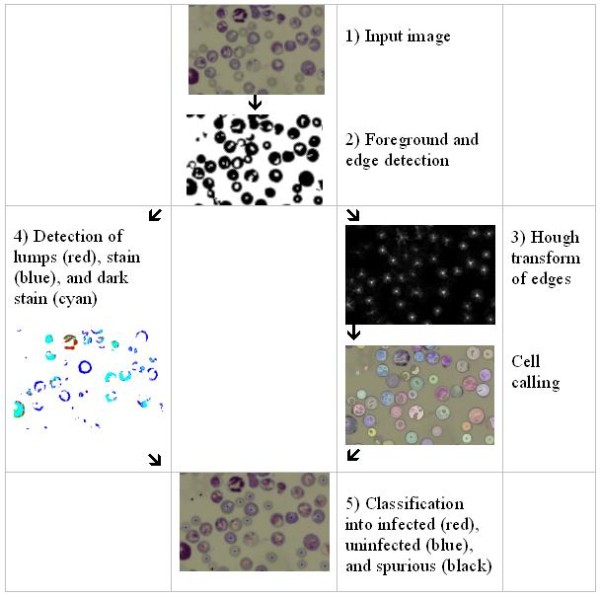
**Flowchart of the automated counting programme "Plasmodium AutoCount"**. Images are first split into pixels to decide on foreground level and detect edges of objects (1 and 2). The process then splits into two paths: determination of the total number of cells via the Hough transform to detect red blood cell edges and detection of parasites by the stain (3 and 4). Finally, total numbers of all cells and infected cells are counted and parasitaemia is calculated (5).

The programme, Plasmodium AutoCount, was written in the Python Programming Language [12] and makes use of the NumPy [13] and SciPy [14] packages for fast numerical calculation. Plasmodium AutoCount has been tested running on the Linux and Windows platforms. The programme is a free software released under the GPL version 2 license. Details of the algorithm can be obtained from the source code [11]. The programme processes all the images in a folder and creates a new folder for all the output images and files. It starts by scaling the image to 680 by 512 pixels for faster processing. Figure 2 is a flowchart of the processing steps.

#### Foreground detection

The first step of processing is to split the image into pixels belonging to cells (or other entities) and background pixels. The image is then enhanced by applying a Gaussian blur of radius 1 to minimize noise. Background variation is reduced by subtracting 75% of a further blurred version of the image. The k-medians algorithm is then used to distinguish pixels belonging to cells from those belonging to the background.

#### Cell detection

Cell detection is by use of a modified version of the circular Hough transform [15] to detect circles of a given size. Background pixels having at least one foreground pixel as one of their eight neighbours, or vice-versa, are classified as edge pixels. The gradient of the image at each edge pixel is estimated using Sobel's operator [16], giving a direction normal to the edge. In the circular Hough transform, pixels one cell radius along the line normal to each edge pixel receive a vote toward being recognized as a cell centre. The "Hough transformed" image is an image representing the number of votes received by each pixel. The circular Hough transform by requiring that a corresponding edge pixel be present one cell diameter along each line was modified. Spurious votes from circles that are not of the desired size were eliminated. It also prevents elliptical cells from producing more than one centre. The transformed images are blurred with a Gaussian blur of radius one pixel. This modified Hough transform is taken for a range of radii (between 5 and 20 pixels), and for each pixel the radius with the maximum votes is found. If a pixel is called as a cell centre, the cell radius is the radius of Hough transform that produce the greatest votes for that pixel. Pixels are called as cell centres in decreasing order of maximum votes received, so long as they are not within 1.25 radii of a pixel receiving greater votes down to a specified minimum number of votes.

#### Infection detection

Parasites appear purple with Giemsa staining. Within this stained region, there are smaller lumps of darker stained material. The criterion for defining infection is the presence of stain spread over a region together with at least one lump. Since the staining is purple, it has the greatest effect on the green channel of the image, so stain and lump detection is performed on this channel. The same approach with different parameters is used for both stain and lump detection. A surrounding average for each pixel is produced, which is a Gaussian kernel weighted average of a given radius, but with only foreground pixels included. Pixels that are darker than a given percentage of the brightness of the surrounding average pixels are flagged. For stain detection, the Gaussian kernel radius used is 10 pixels. For lump detection, the Gaussian kernel radius is 3 pixels. As a measure of the non-localization of staining in a cell, the average location of all the stained pixels in a cell was evaluated, and then the mean squared distance of stained pixels from this average location was worked out. A cell with this mean squared distance exceeding 3 and at least one lump pixel is regarded as infected.

#### Spurious hit filtering

The Hough transform produces spurious hits to ruptured cells, debris, and white blood cells (WBC), which should be excluded from counting. Accordingly, Hough transform-detected "cells" are filtered if they are smaller than a 9 pixels, or the center of mass of foreground pixels was greater than a certain distance from the Hough transform determined centre, or contained greater than a certain proportion of darkly stained pixels (usually WBCs).

#### Statistical analyses

Parasitaemia totals obtained by manual or automated counting were compared using Pearson's correlation test. The coefficient of variations was also expressed as root-mean-square (RMS) using the following formula: RMS = $\sqrt{\frac{\sum {\left(x1-x2\right)}^{2}}{n}}$, where ×1 and ×2 are two separate readings and n is the total number of counted smears. All analyses, apart from RMS calculation, were performed in Graphpad Prism 5.

## Results

### Manual counting

Before the calibration of Plasmodium AutoCount programme, parasitaemia of selected images were counted manually using the Manual Counting Aid described in the Materials and Methods Section.

### Programme calibration

**Figure 3:**
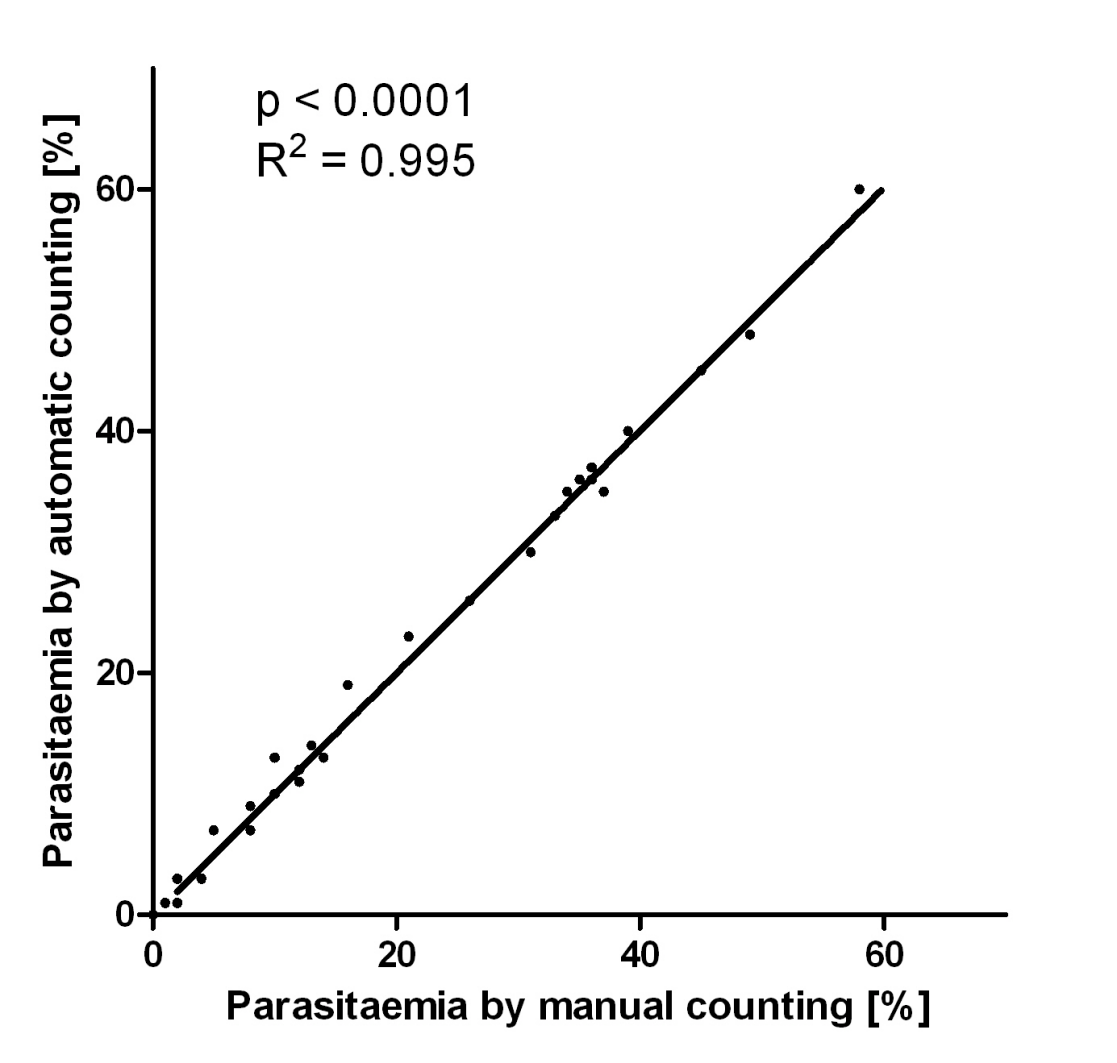
**Correlation between automated and manual counting for 32 standard images**. The straight line indicates perfect matches. R^2 ^and p values are obtained from the Pearson's test.

**Figure 4:**
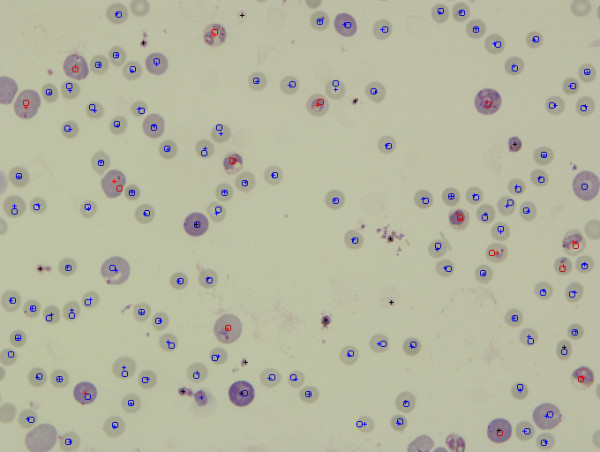
**An example of the output of the automated counting programme compared to manual counting results**. Crosses represent cells counted by Plasmodium AutoCount whereas squares represent cells counted by manual counting. Uninfected cells are marked (crosses or squares) in blue colour whereas infected cells are marked in red. Black crosses indicate filtered debris.

The algorithm for the Plasmodium AutoCount programme is outlined in Figure 2. Values for threshold levels of various parameters were set using a number of standardized images. Thirty-two images were selected for this purpose from a previous mouse challenge experiment, with parasitaemia determined by trained personnel ranging from 0% to 58%. For each image, the difference between parasitaemia values derived from manual or automated counting was expressed as a RMS value. The aim of the calibration process was to adjust the parameters so that a minimum RMS value was obtained. After adjustment through optimizing the parameters, a minimum RMS value of 1.3 was achieved; this represented the lowest possible difference and standard deviation obtainable between manual and automated counting. As shown in Figure 3, the parasitaemia determined using these values to calibrate the programme was highly correlated with those determined by manual counting (R^2 ^= 0.995, p < 0.0001). Figure 4 shows a typical output image comparing the automated and manual counting results. Cell density is somewhat sparser than a typical smear to allow easy examination of the programme output.

### Comparison between automated and manual counting

**Figure 5:**
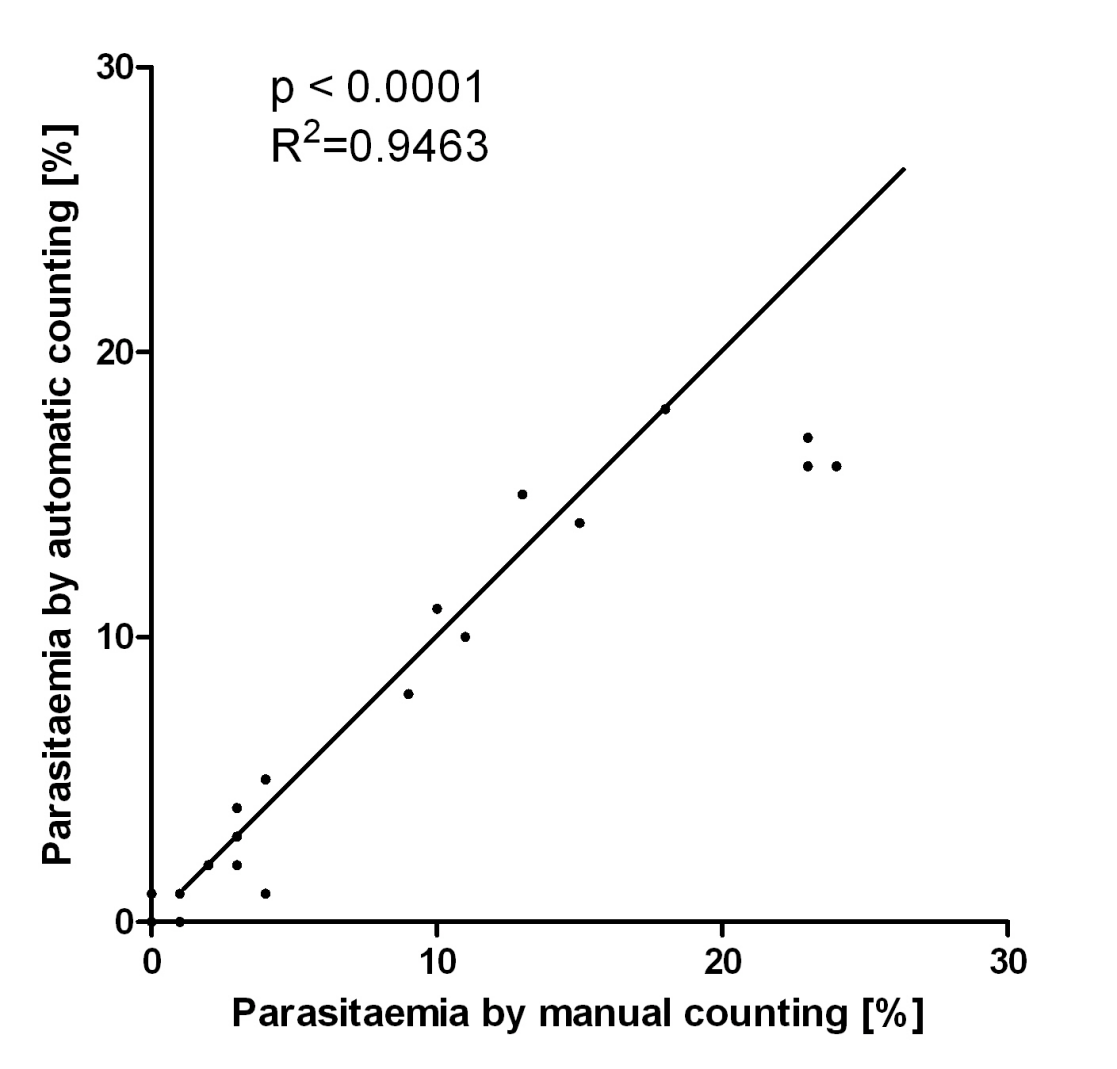
**Correlation between automated and manual counting for the images collected at day 7 post-challenge**. The straight line indicates perfect matches. R^2 ^and p values are obtained from the Pearson's Test.

To assess the accuracy of the calibrated programme, a mouse challenge experiment was performed in which twenty mice were immunized with PyMSP1_19_, and five mice were immunized with PBS as controls. All of the mice were infected with *P. yoelii *parasites, and daily blood samples from each mouse were taken and smears made from day 3 to day 19 after infection. The immunized and control mice showed different levels of parasitaemia. A total of 284 blood smear images were collected and subjected to the automated counting programme, as well as being counted manually by an experienced experimenter. The correlation plot in Figure 5 summarized the relationship between the manually and automatically obtained parasitaemia estimates for smears made at day 7 post-infection and shows a very high correlation (R^2 ^= 0.9463, p < 0.0001). The performance was achieved consistently for images at different stages of infection and was independent of the actual parasitaemia level. The correlation graphs from day 5 to day 17 are shown in Additional file 1, Figure S1-S12, and the R^2 ^and RMS values are summarized in Table 1. Intra-assay variability of the programme was determined by analysing the same set of images three times using Plasmodium AutoCount. Results were identical between analyses showing the method is highly reproducible.

**Table 1:** Correlation and variation between manual and automated counting results from a challenge infection experiment

	**R**^**2**^	RMS
**Day 5**	0.7808	0.4

**Day 6**	0.8759	1.0

**Day 7**	0.9463	1.4

**Day 8**	0.9813	1.2

**Day 9**	0.9644	1.2

**Day 10**	0.8204	1.5

**Day 11**	0.8986	1.7

**Day 12**	0.9586	1.4

**Day 13**	0.9513	1.4

**Day 14**	0.7804	1.3

**Day 15**	0.9369	0.7

**Day 17**	0.7671	0.8

The R^2 ^values are obtained using Pearson's test, all p values are < 0.0001. RMS values are the root-mean-square values of the differences.

Two potential confounding factors in parasitaemia determination are the presence of WBCs and reticulocytes. To investigate whether the presence of WBC affects the accuracy of Plasmodium AutoCount, all the images for Day 3-6 that contained WBCs were chosen and the number of WBCs identified as infected cells (false positives) was determined. The results are shown in Additional file 2, Table S1. Out of 33 WBCs which should ideally be excluded, the programme regarded only one of them as infected. However, in nine other cases, uninfected red cells clumping around a central WBC were scored as infected. An example of this is shown in the Additional file 1, Figure S13.

The ability of Plasmodium AutoCount to differentiate between infected and uninfected reticulocytes was investigated. Images from Day 12 were chosen as there would be a substantial proportion of reticulocytes due to loss of RBC. The results are shown in Additional file 2, Table S2. Reticulocytes are classified manually as RBCs with more purple colour and often with slightly larger diameters. For uninfected reticulocytes, the identification by the programme is relatively easy. However, the identification of infected reticulocytes is harder as infected RBCs are quite often swollen and the colour also appears darker partly due to the presence of the parasite. The results indicated that the programme is relatively good at identifying uninfected reticulocytes as uninfected cells. However, there is a higher false negative rate in which infected reticulocytes were regarded as uninfected.

### Variations in manual counting

Although the level of correlation was high, it was not perfect and the question of whether this was likely to be a significant defect in this approach was raised. Since parasitemia estimation is typically performed by different members of a laboratory, it was set out to determine whether the degree of difference between the results from the programme and those of a trained observer would be equivalent to differences seen among different trained experimenters. A set of 100 images, half from day 4 smears and the other half from day 10 smears of the mouse challenge experiment, were independently counted by five qualified researchers with experience in parasitaemia estimation. Each of these researchers has worked on malaria for at least three years and has had extensive experience scoring smears. Table 2 shows the R^2 ^and RMS values of the parasitaemia data obtained. The manually counted parasitaemia exhibited R^2 ^values between 0.9867 and 0.9951, and RMS values between 1.1 and 4.3, which represents the differences in human decision making. Of note, the differences between individuals and Plasmodium AutoCount are comparable to the variations among individuals as R^2 ^values are between 0.9742 and 0.9860, and RMS values range from 2.1 to 3.1.

**Table 2:** Correlation and variation of manual counting results between different examiners as compared to automated counting

	Manual Counting								Automated Counting	
			
	P2		P3		P4		P5			
	
	**R**^**2**^	RMS	**R**^**2**^	RMS	**R**^**2**^	RMS	**R**^**2**^	RMS	**R**^**2**^	RMS
**P1**	0.9940	3.8	0.9883	4.3	0.9890	3.1	0.9951	1.1	0.9860	2.5

**P2**			0.9931	1.1	0.9912	1.6	0.9925	3.6	0.9824	2.9

**P3**					0.9916	1.8	0.9867	4.1	0.9805	3.1

**P4**							0.9879	2.9	0.9742	2.1

**P5**									0.9848	2.6

The R^2 ^values are obtained using Pearson's test, all p values are < 0.0001. RMS values are the root-mean-square values of the differences. P1 to P5 represent the five experimenters who counted the slides.

## Discussion

Despite advances in imaging technology, manual microscopic enumeration of Giemsa-stained blood smears remains the most widely and commonly used method for *Plasmodium *parasitaemia determination, particularly in the study of model infections. This is a time-consuming and tiring process that can be significantly affected by the expertise of the observer and has variable accuracy. An automated image analysis system that can be used for fast, accurate, reproducible and reliable determination of parasitaemia would be a worthwhile advance [1]. Several automated image-processing approaches for blood smear analysis have been attempted with some reported success. For example, an automated image processing programme has been developed by Ross *et al *for the diagnosis and classification of *Plasmodium *species [6], which reported a sensitivity of 85% and a positive predictive value of 81%. An image analysis-based programme, named MalariaCount, was reported to provide rapid and accurate determination of parasitaemia for blood smears of *in vitro P. falciparum *culture material [8]. No programme has been available for automated determination of parasitaemia from mouse challenge experiments. During a challenge infection, blood samples from a large number of mice are generally required to be counted on a daily basis and, in some cases, results are needed on the same day in order to make decision as to whether to sacrifice the mice. Proudfoot *et al *have reported a partial automation approach for counting infected mouse blood smears; however completely automated scoring remained elusive [9].

An image analysis programme, Plasmodium AutoCount, for the routine determination of percent-parasitaemia in thin blood smears from *Plasmodium yoelii*-infected mice was developed. The programme has also proved useful for analysis of samples taken from subjects at different stages of infection, with various levels of parasitaemia. The parasitaemia values generated automatically are highly correlated with those determined by manual counting, and the differences between them are comparable to those observed among different examiners. The procedure is rapid, and the time-saving is significant. About 100 images can be processed in half an hour using a standard desktop computer, in contrast to manual counting of these smears which would take about six hours. The programme was subsequently used to monitor parasitaemia from a total of 174 mice in four challenge experiments. Parasitaemia from up to 50 mice have been measured on a daily basis, and manual counting of randomly selected smears confirmed the accuracy of the automatically generated parasitaemia values.

Plasmodium AutoCount does not recognise the morphology of parasites; instead it detects the darkness levels of the images and identifies images that occupy a certain proportion of the whole cell. Its accuracy relies on well-prepared blood smears. Clean, evenly-stained smears containing separated cells with few lysed cells are necessary. The quality of photographs is also important, with sharply focused, well-illuminated images required. In reality, smear images can be far from optimal, such as the presence of clustered cells, WBCs or dead parasites, as well as colour variation due to differing incubation times with staining solution. These factors will significantly affect the results generated by Plasmodium AutoCount. In these situations, a hybrid method for semi-automated parasitaemia determination was suggested. Firstly the total number of cells is counted using Plasmodium AutoCount, then the infected cells are counted using the Cell Counting Aid. This method could be used to overcome any inaccuracy of the automated counting programme for some poorly prepared or irregular smears.

Two possible causes of significant error in parasitaemia estimation are the presence of WBCs and reticulocytes. As noted above WBCs are quite accurately excluded, but on occasions surrounding uninfected RBCs may be incorrectly judged to be infected. It is suggested that obvious clumping within an image be a criterion for not using that particular field for automated counting. If clumping is unavoidable, then given the ratio of white cells to red cells early in the course of murine infection, this would lead to an overestimation of parasitaemia of at most 0.03%. During the course of most infections, this is unlikely to be a significant problem in interpretation but may prevent cases of sterile protection being recognized. In this case, manual re-examination of the scored pictures would allow the investigator to arrive at the correct conclusion. A possible future modification to address this would be to build in the capacity for the software to recognize a clump and not interpret it as red cell cytoplasm surrounding an area of stain, an appearance similar to a parasitized cell.

With respect to reticulocytes, the problem is incorrect classification of infected reticulocytes as uninfected. Another version of the software was developed that gives more balanced error rates, but at present neither false positive or false negative reticulocyte rates can be corrected because altering the parameters to correct one problem, increases the reverse problem. This remains an ongoing area of study. The point to emphasize though is that these errors also occur among human observers and overall, the programme is very similar to the average counts obtained by manual counting, but with significantly less time and work and no requirement for experienced personnel together with the advantage of a permanent record of how the value was obtained.

Although Plasmodium AutoCount was calibrated on *P. yoelii*-infected mouse blood smears in its current version, the programme can potentially be extended to estimate parasitaemia from infected mouse, primate and even human blood smears, which may involve other *Plasmodium *species, by adjusting the parameters that set the threshold level for detection. It might also be adjusted to determine parasitaemia in cultured blood samples for *in vitro *experiments such as drug susceptibility tests and growth inhibition assays.

## Conclusions

Plasmodium AutoCount has proven to be a useful tool for rapid and accurate determination of parasitaemia from infected mouse blood. The parasitaemia values are highly correlated with those determined by manual counting, and the variations between them are comparable to those observed among different examiners. The programme can be expanded to estimate parasitaemia from infected human blood as well as *in vitro *cultured infected blood samples.

## Competing interests

The authors declare that they have no competing interests.

## Authors' contributions

CM prepared the slides, collected the data and performed all analyses; PH wrote the programme and participated in the calibration; LW involved in project design and testing throughout the project; RC designed the project and initiated the idea. All authors have read and approved the final manuscript.

## Supplementary Material

Additional file 1 **Supplementary Figures**. The Powerpoint file contains Figure S1 to Figure S13. Figure S1 Correlation graph of manual and automatic counting results for day 5. Figure S2 Correlation graph of manual and automatic counting results for day 6. Figure S3 Correlation graph of manual and automatic counting results for day 7. Figure S4 Correlation graph of manual and automatic counting results for day 8. Figure S5 Correlation graph of manual and automatic counting results for day 9. Figure S6 Correlation graph of manual and automatic counting results for day 10. Figure S7 Correlation graph of manual and automatic counting results for day 11. Figure S8 Correlation graph of manual and automatic counting results for day 12. Figure S9 Correlation graph of manual and automatic counting results for day 13. Figure S10 Correlation graph of manual and automatic counting results for day 14. Figure S11 Correlation graph of manual and automatic counting results for day 15. Figure S12 Correlation graph of manual and automatic counting results for day 17. Figure S13 Example image of uninfected red blood cell scored as infected when it is too close to a white blood cell. Click here for file

Additional file 2 **Supplementary Tables**. The Word document contains Table S1 and Table S2. Table S1 The influence of white blood cells on the accuracy of Plasmodium AutoCount. Table S2 The influence of reticulocytes on the accuracy of Plasmodium AutoCount. Click here for file
