# Supplementary material for: Automated estimation of parasitaemia of Plasmodium yoelii-infected mice by digital image analysis of Giemsa-stained thin blood smears
Source: Malar J. 2010 Dec 1;9:348. doi: 10.1186/1475-2875-9-348 (PMC3245511; doi:10.1186/1475-2875-9-348)
Supplement: Additional file 1 — Supplementary Figures. The Powerpoint file contains Figure S1 to Figure S13. Figure S1 Correlation graph of manual and automatic counting results for day 5. Figure S2 Correlation graph of manual and automatic counting results for day 6. Figure S3 Correlation graph of manual and automatic counting results for day 7. Figure S4 Correlation graph of manual and automatic counting results for day 8. Figure S5 Correlation graph of manual and automatic counting results for day 9. Figure S6 Correlation graph of manual and automatic counting results for day 10. Figure S7 Correlation graph of manual and automatic counting results for day 11. Figure S8 Correlation graph of manual and automatic counting results for day 12. Figure S9 Correlation graph of manual and automatic counting results for day 13. Figure S10 Correlation graph of manual and automatic counting results for day 14. Figure S11 Correlation graph of manual and automatic counting results for day 15. Figure S12 Correlation graph of manual and automatic counting results for day 17. Figure S13 Example image of uninfected red blood cell scored as infected when it is too close to a white blood cell. [file 1475-2875-9-348-S1.PPTX]

## Slide 1
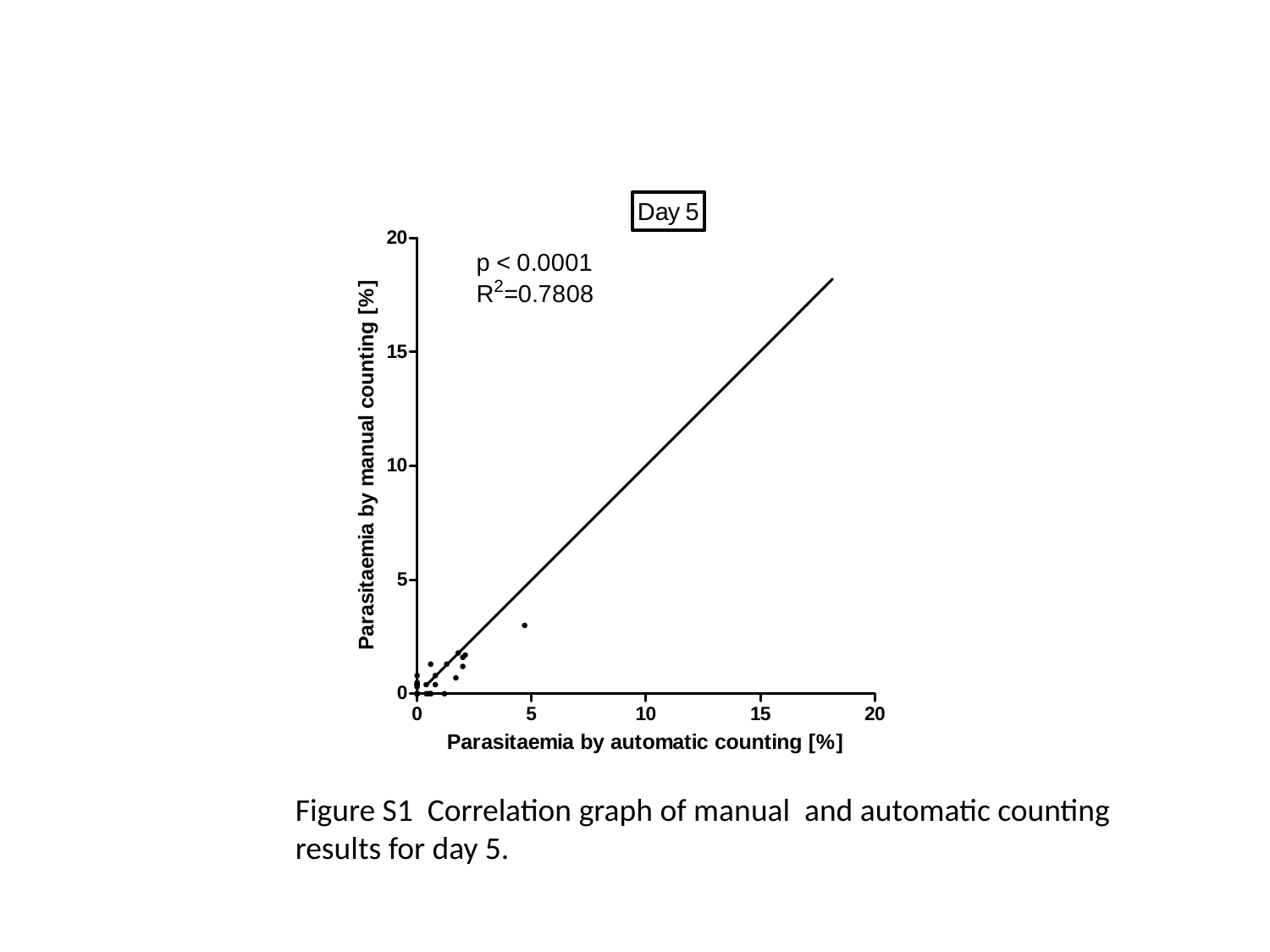

Figure S1 Correlation graph of manual and automatic counting
results for day 5.

## Slide 2
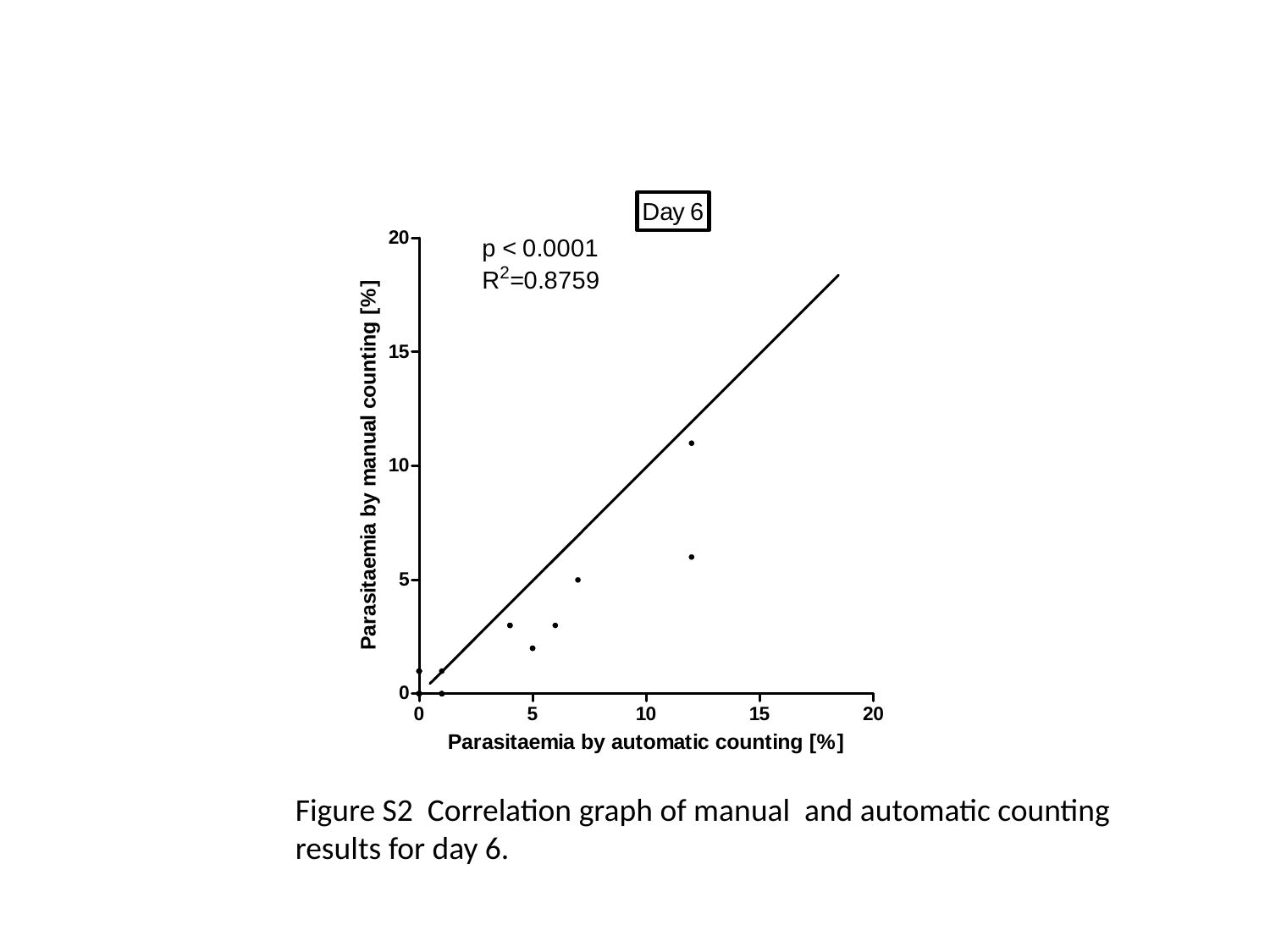

Figure S2 Correlation graph of manual and automatic counting
results for day 6.

## Slide 3
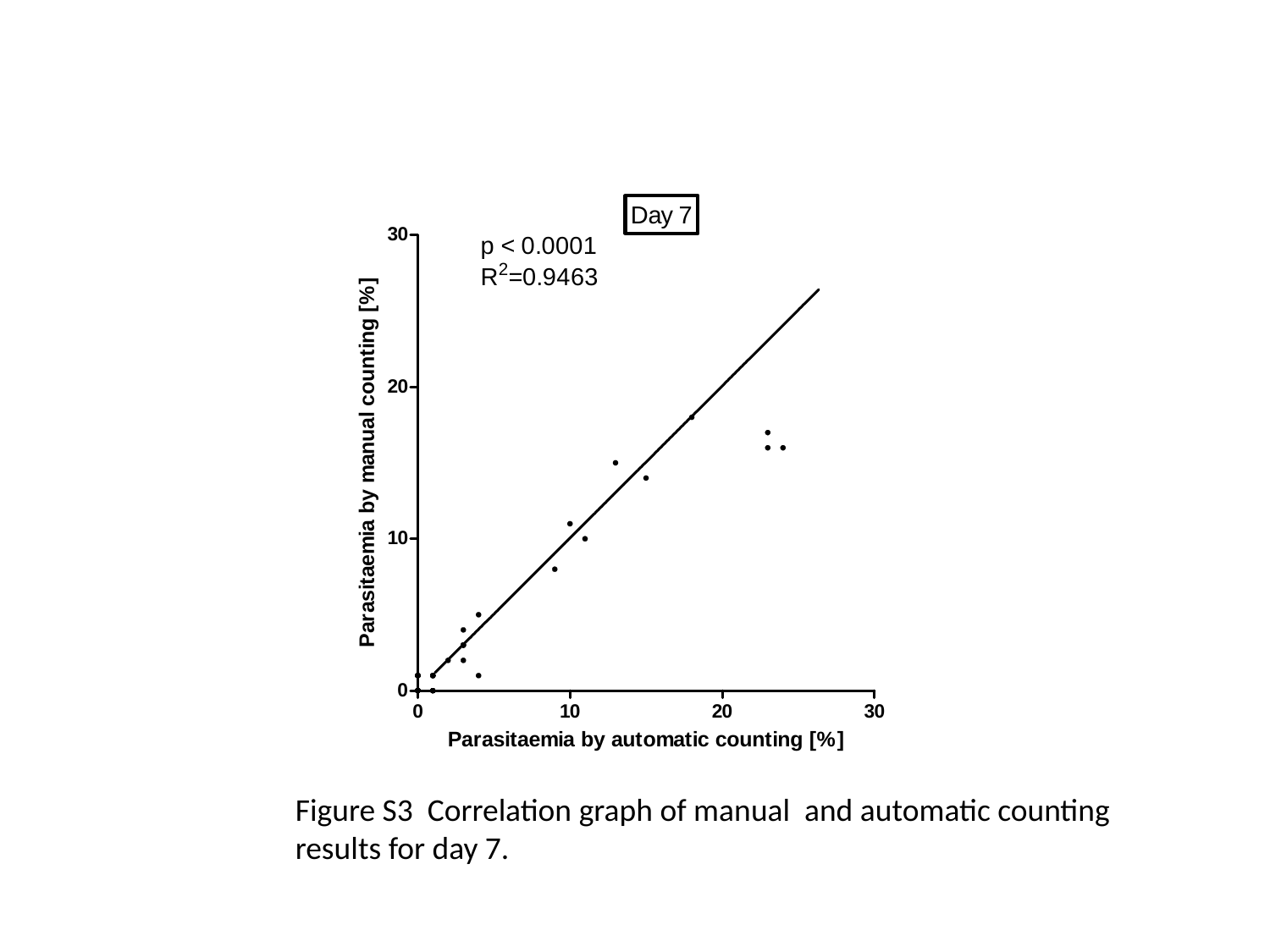

Figure S3 Correlation graph of manual and automatic counting
results for day 7.

## Slide 4
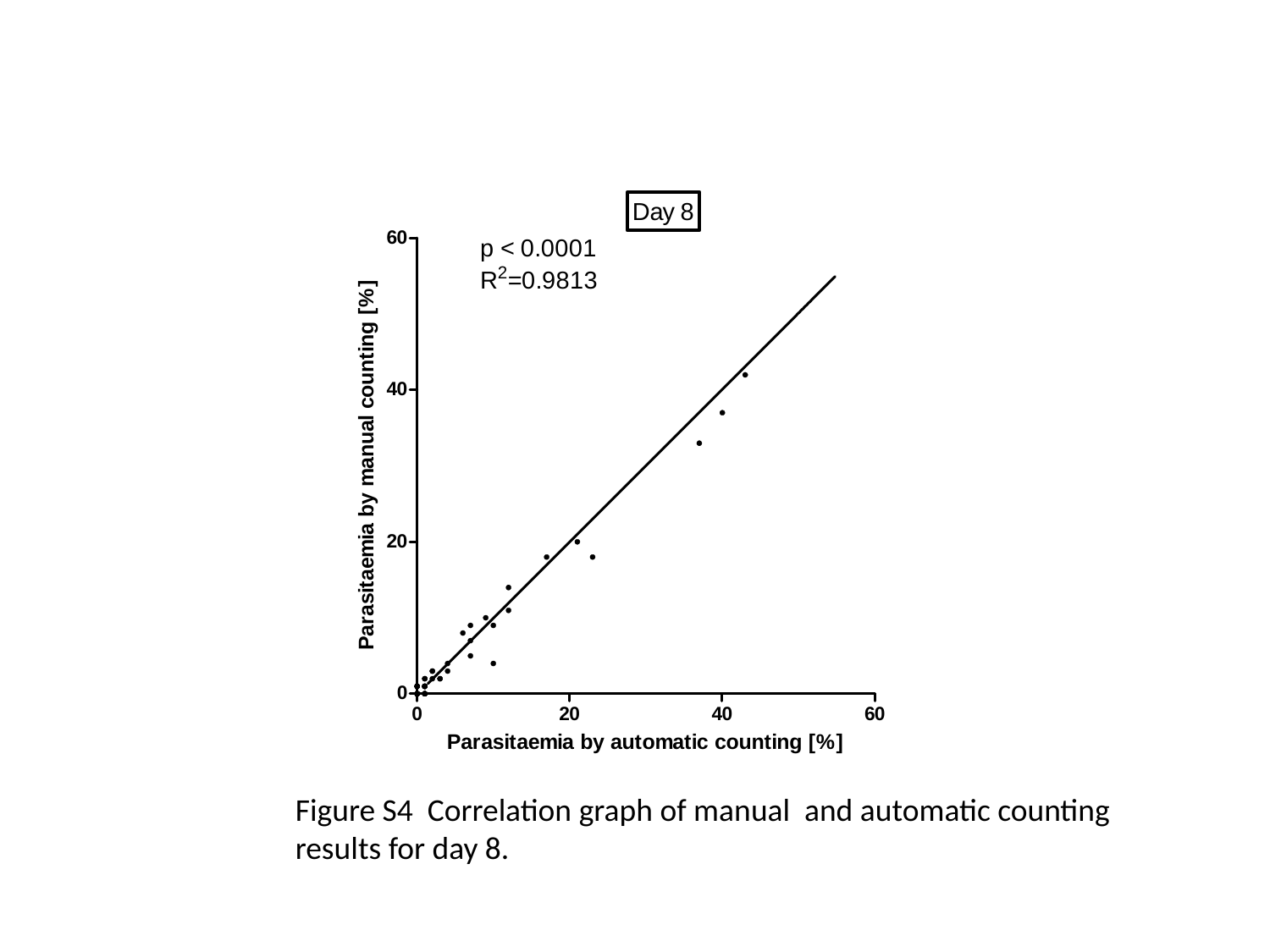

Figure S4 Correlation graph of manual and automatic counting
results for day 8.

## Slide 5
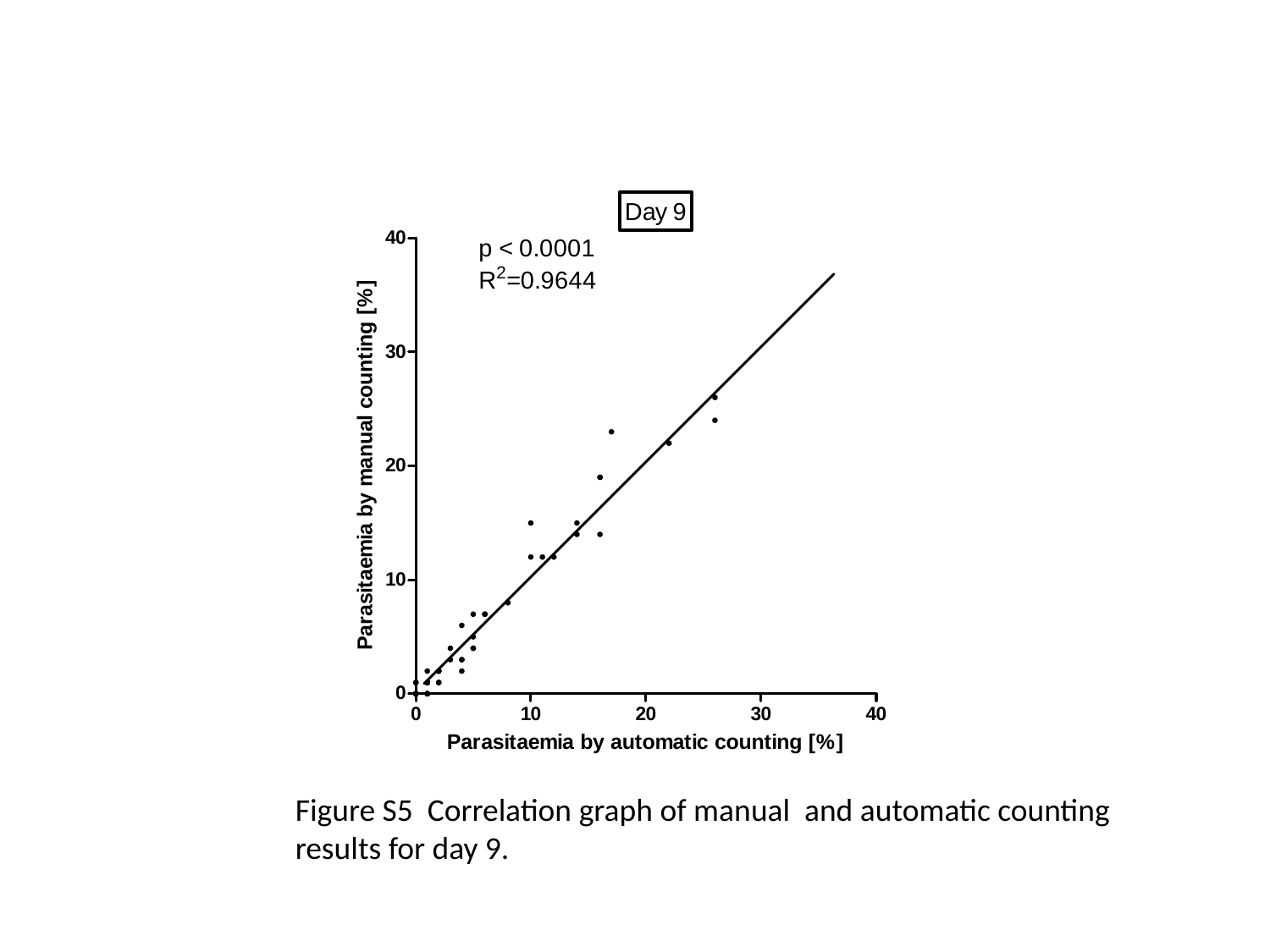

Figure S5 Correlation graph of manual and automatic counting
results for day 9.

## Slide 6
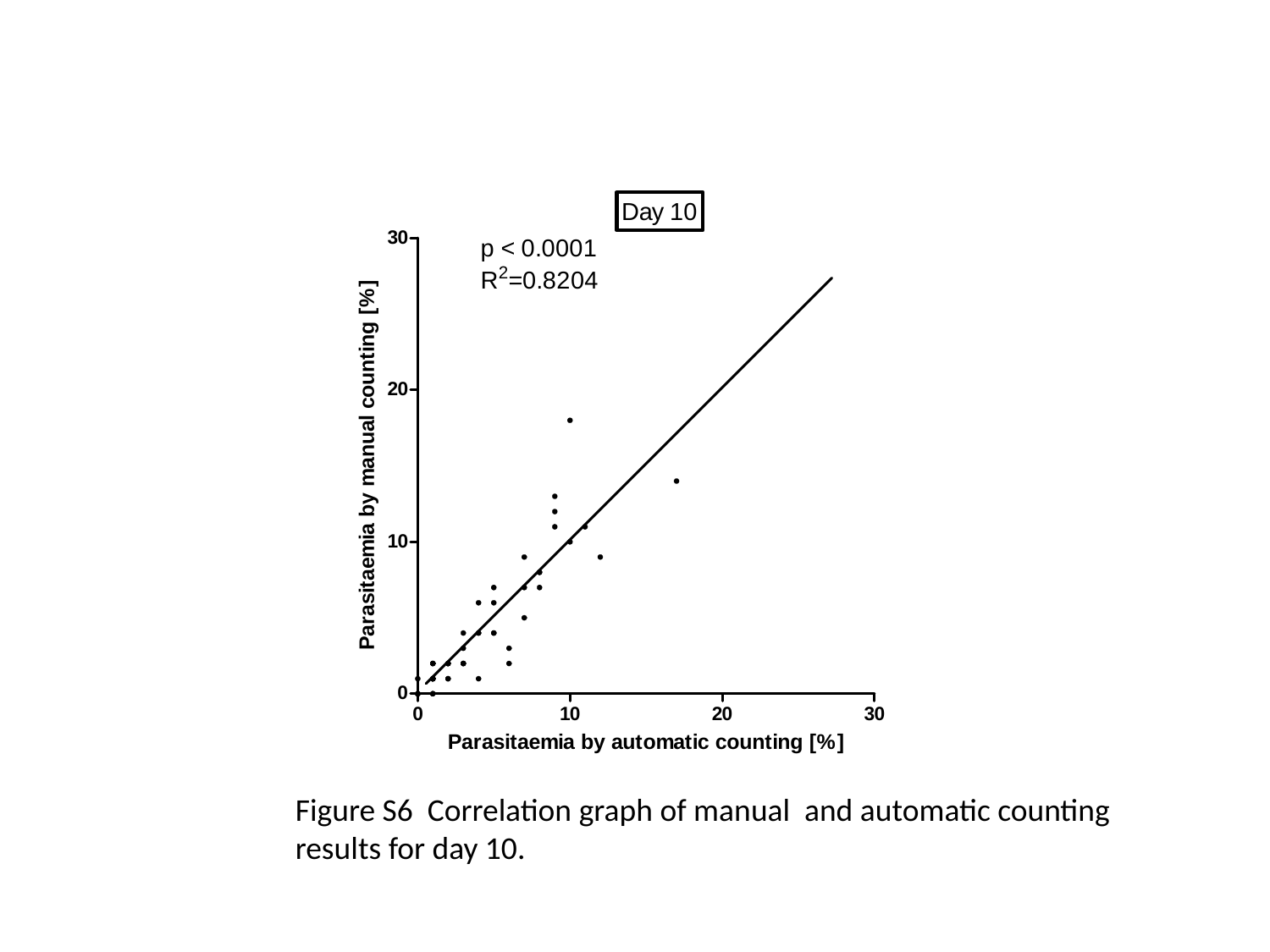

Figure S6 Correlation graph of manual and automatic counting
results for day 10.

## Slide 7
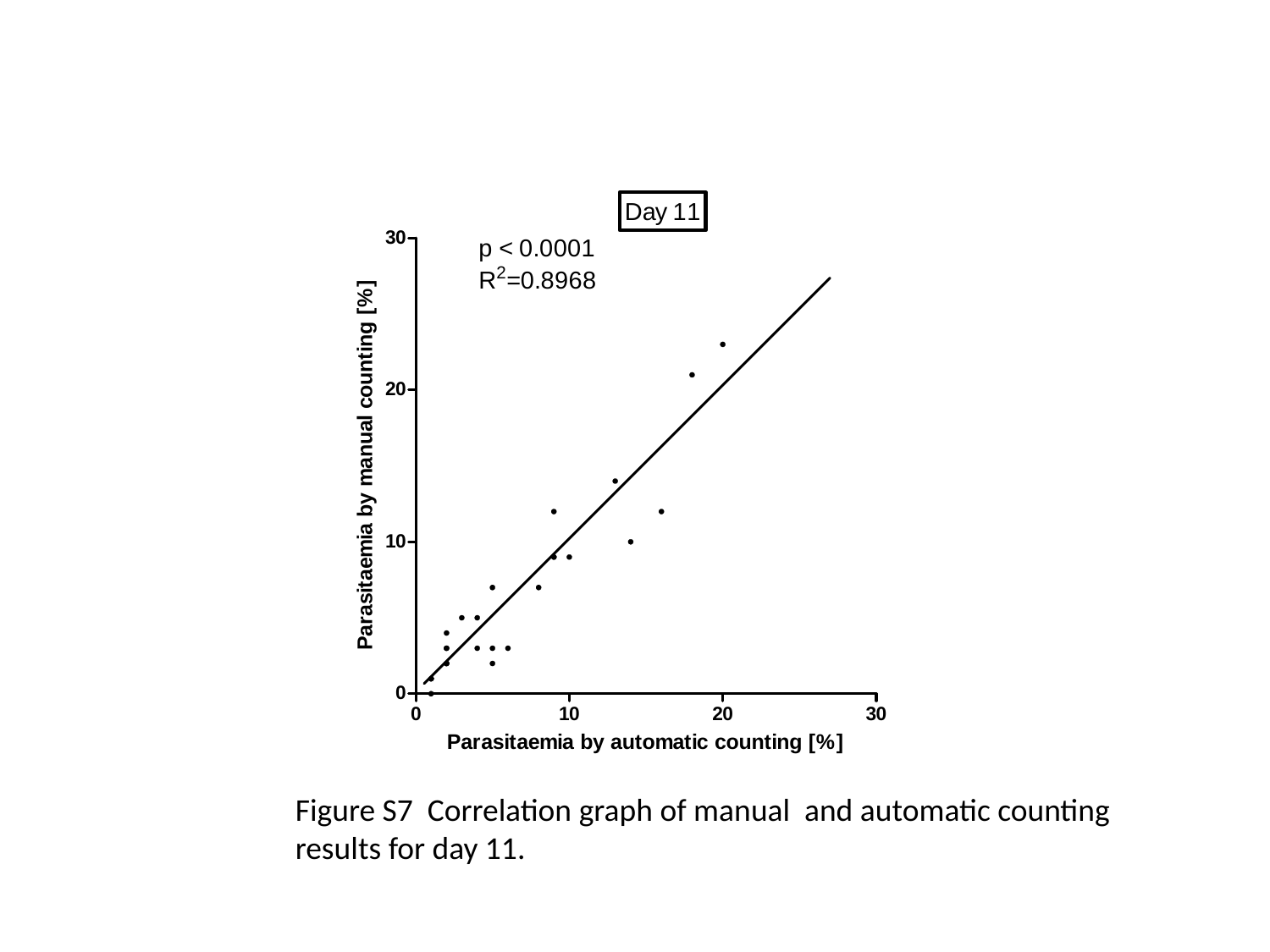

Figure S7 Correlation graph of manual and automatic counting
results for day 11.

## Slide 8
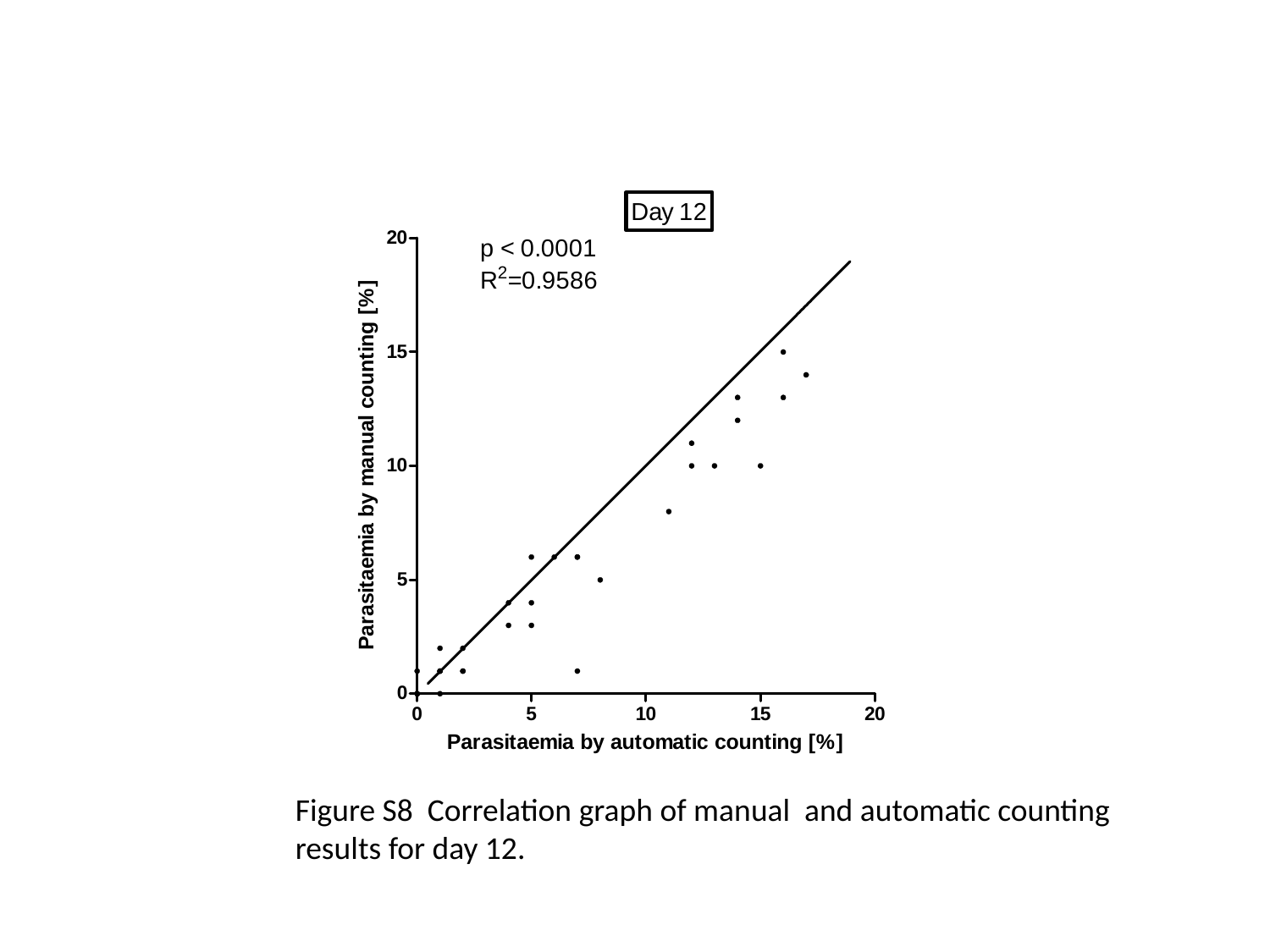

Figure S8 Correlation graph of manual and automatic counting
results for day 12.

## Slide 9
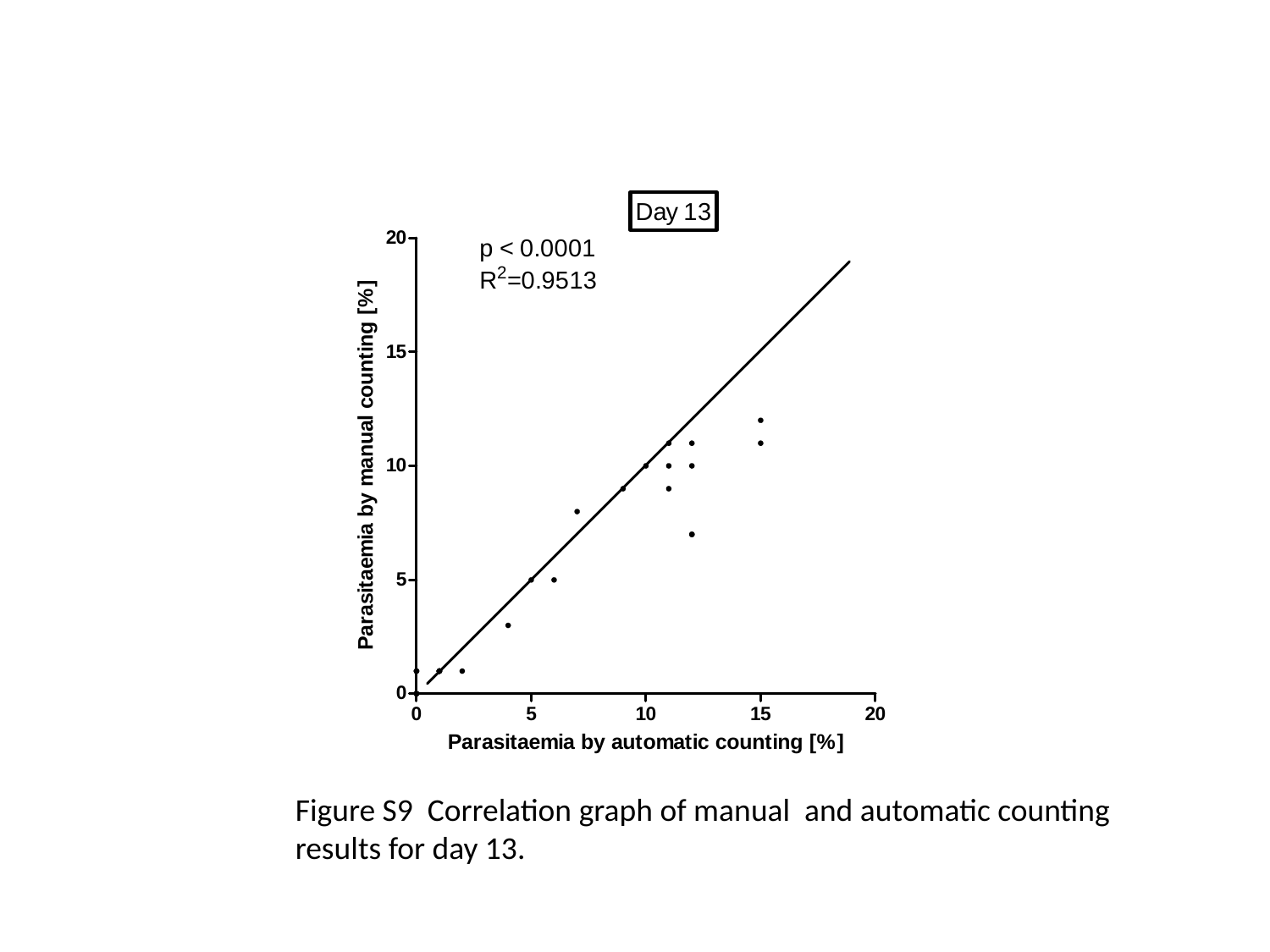

Figure S9 Correlation graph of manual and automatic counting
results for day 13.

## Slide 10
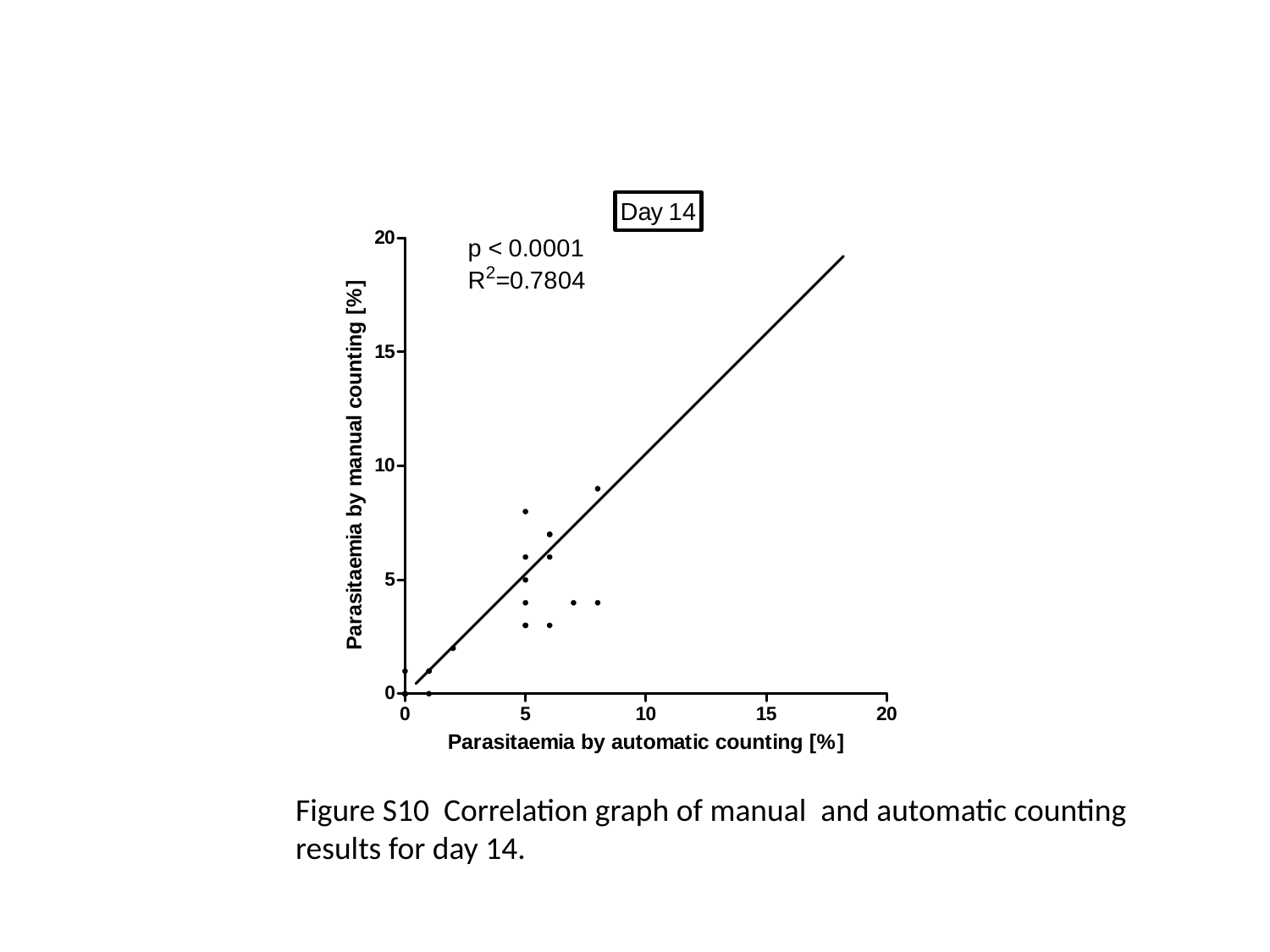

Figure S10 Correlation graph of manual and automatic counting
results for day 14.

## Slide 11
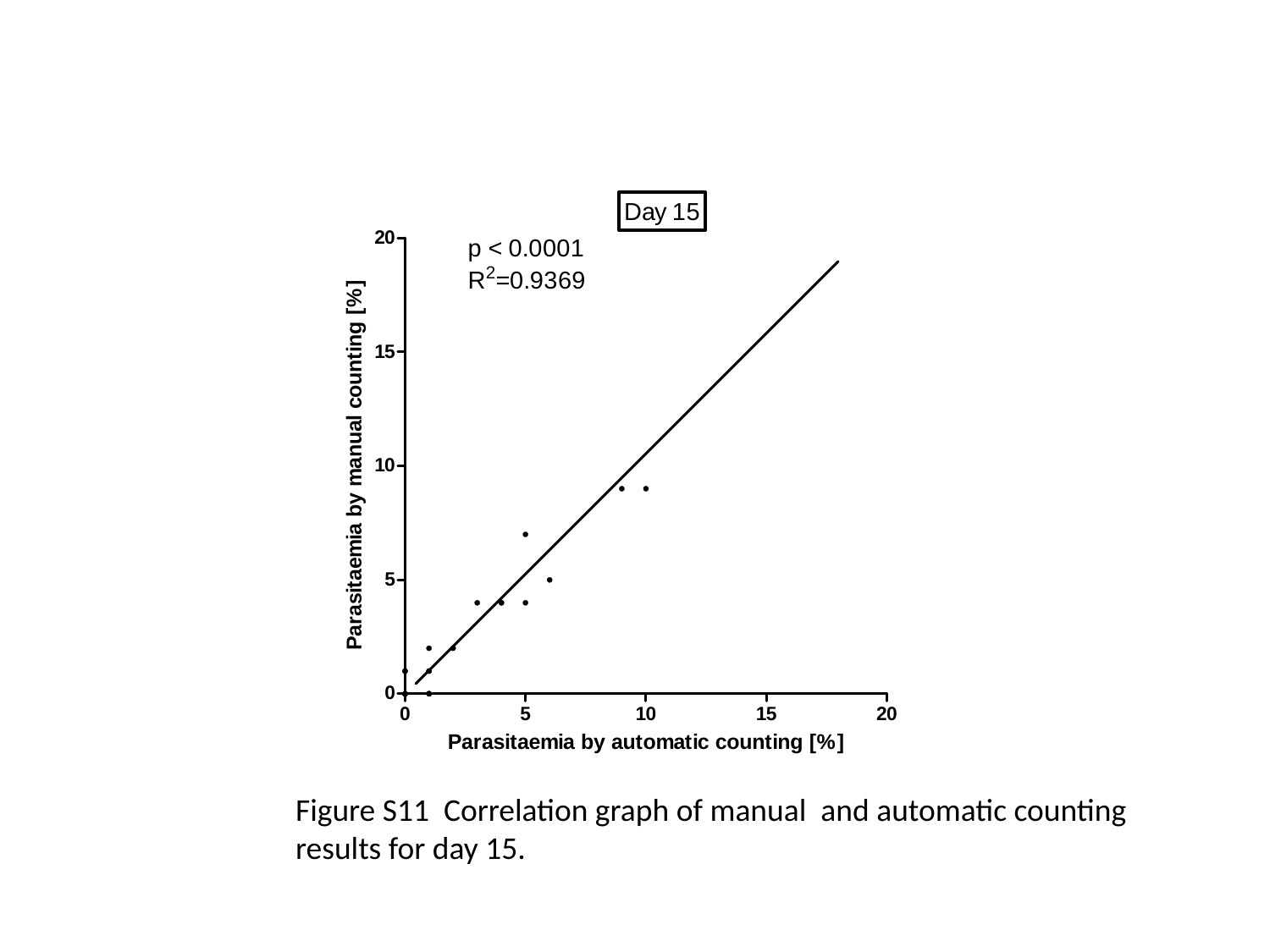

Figure S11 Correlation graph of manual and automatic counting
results for day 15.

## Slide 12
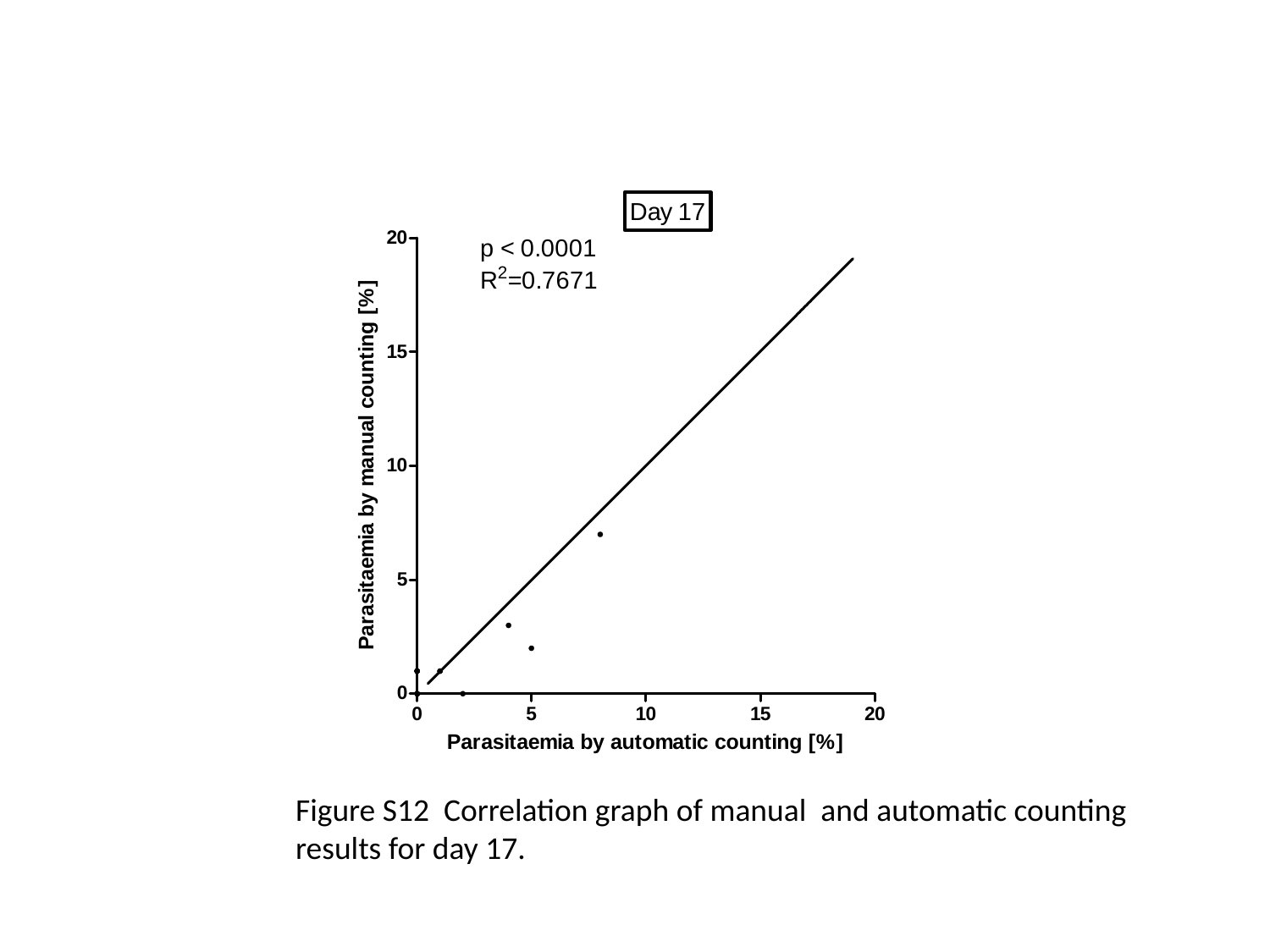

Figure S12 Correlation graph of manual and automatic counting
results for day 17.

## Slide 13
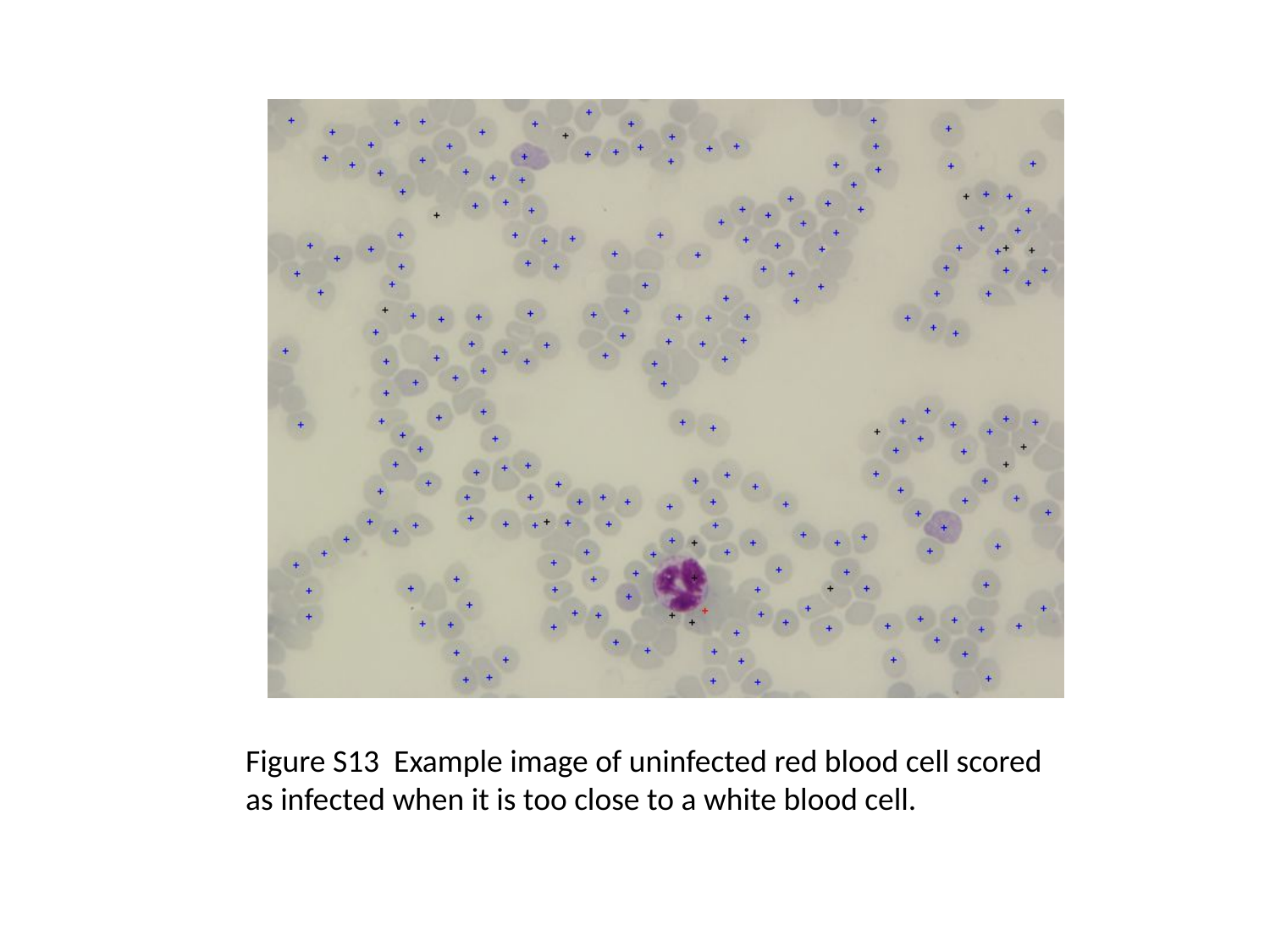

Figure S13 Example image of uninfected red blood cell scored
as infected when it is too close to a white blood cell.
