# Supplementary material for: Automated estimation of parasitaemia of Plasmodium yoelii-infected mice by digital image analysis of Giemsa-stained thin blood smears
Source: Malar J. 2010 Dec 1;9:348. doi: 10.1186/1475-2875-9-348 (PMC3245511; doi:10.1186/1475-2875-9-348)
Supplement: Additional file 2 — Supplementary Tables. The Word document contains Table S1 and Table S2. Table S1 The influence of white blood cells on the accuracy of Plasmodium AutoCount. Table S2 The influence of reticulocytes on the accuracy of Plasmodium AutoCount. [file 1475-2875-9-348-S2.DOCX]

**Table S1 The influence of white blood cells on the accuracy of Plasmodium AutoCount.**

| **Day** | **Image** | **Total cells** | **Total WBC** | **False +ve** | **Clumping** |
| --- | --- | --- | --- | --- | --- |
| 3 | A1#1c | 156 | 1 | 0 | No |
| 3 | A1#2a | 209 | 1 | 3 | Yes |
| 3 | A1#2b | 156 | 2 | 0 | No |
| 3 | A1#3a | 170 | 1 | 0 | Yes |
| 3 | A2#1b | 183 | 1 | 0 | No |
| 3 | A2#2c | 222 | 1 | 0 | No |
| 3 | A2#3c | 293 | 1 | 0 | No |
| 3 | B2#5c | 303 | 1 | 1 | Yes |
| 3 | C#1c | 250 | 1 | 0 | No |
| 3 | C#3c | 266 | 1 | 0 | No |
| 3 | C#4c | 235 | 1 | 0 | Yes |
| 4 | A1#1c | 164 | 2 | 2 | Yes |
| 4 | A1#2b | 197 | 1 | 0 | No |
| 4 | A1#3c | 156 | 1 | 0 | No |
| 4 | A1#5a | 230 | 1 | 0 | Yes |
| 4 | A2#1c | 272 | 1 | 0 | No |
| 4 | B1#2c | 164 | 1 | 0 | No |
| 4 | B1#3c | 276 | 1 | 0 | No |
| 5 | A2#2b | 215 | 1 | 0 | No |
| 5 | A2#3a | 247 | 2 | 1 | Yes |
| 5 | A2#4c | 224 | 1 | 0 | No |
| 5 | B1#4a | 227 | 1 | 1 | Yes |
| 5 | B2#2a | 238 | 1 | 1 | No |
| 5 | C#1a | 235 | 1 | 0 | No |
| 5 | C#4b | 306 | 1 | 1 | Yes |
| 5 | C#4c | 331 | 1 | 0 | No |
| 6 | A1#1c | 178 | 2 | 0 | Yes |
| 6 | A2#3b | 189 | 1 | 0 | No |
| 6 | B2#3b | 177 | 1 | 0 | No |
|  | **Total** | **6469** | **33** | **10** |  |

Plasmodium AutoCount has the ability to differentiate white blood cells (WBCs) from red blood cells (RBCs) . Normally, WBCs are excluded but there can be false positive cases. Most of these cases were due to clumping of RBCs with the WBCs.

**Table S2 The influence of reticulocytes on the accuracy of Plasmodium AutoCount.**

| **Image** | **RBC** | **pRBC** | **Parasitaemia** | **Reticulocyte** | **Infected Reti** | **False +ve** | **False -ve** |
| --- | --- | --- | --- | --- | --- | --- | --- |
| A1#1a | 179 | 18 | 10% | 76 | 22 | 0 | 4 |
| A1#1b | 171 | 22 | 13% | 64 | 17 | 0 | 2 |
| A1#1c | 193 | 28 | 15% | 72 | 12 | 2 | 3 |
| A1#2a | 148 | 8 | 5% | 48 | 10 | 0 | 6 |
| A1#2b | 120 | 12 | 10% | 46 | 16 | 0 | 4 |
| A1#2c | 147 | 1 | 1% | 50 | 10 | 0 | 9 |
| A1#3a | 156 | 1 | 1% | 35 | 3 | 0 | 2 |
| A1#3b | 162 | 1 | 1% | 35 | 1 | 0 | 0 |
| A1#3c | 142 | 2 | 1% | 32 | 3 | 0 | 2 |
| A1#4a | 161 | 1 | 1% | 8 | 1 | 0 | 0 |
| A1#4b | 241 | 1 | 0% | 13 | 0 | 0 | 0 |
| A1#4c | 248 | 0 | 0% | 8 | 0 | 0 | 0 |
| A1#5a | 225 | 0 | 0% | 7 | 0 | 0 | 0 |
| A1#5b | 229 | 0 | 0% | 9 | 0 | 0 | 0 |
| A1#5c | 231 | 0 | 0% | 5 | 0 | 0 | 0 |
| A2#1a | 161 | 0 | 0% | 3 | 0 | 0 | 0 |
| A2#1b | 181 | 0 | 0% | 0 | 0 | 0 | 0 |
| A2#1c | 175 | 0 | 0% | 5 | 0 | 0 | 0 |
| A2#2a | 262 | 0 | 0% | 0 | 0 | 0 | 0 |
| A2#2b | 293 | 0 | 0% | 4 | 0 | 0 | 0 |
| A2#2c | 304 | 0 | 0% | 5 | 0 | 0 | 0 |
| A2#3a | 217 | 0 | 0% | 0 | 0 | 0 | 0 |
| A2#3b | 261 | 0 | 0% | 3 | 0 | 0 | 0 |
| A2#3c | 243 | 0 | 0% | 2 | 0 | 0 | 0 |
| A2#4a | 203 | 13 | 6% | 23 | 13 | 0 | 2 |
| A2#4b | 195 | 11 | 6% | 15 | 5 | 0 | 0 |
| A2#4c | 198 | 11 | 6% | 12 | 9 | 0 | 2 |
| A2#5a | 289 | 0 | 0% | 8 | 0 | 0 | 0 |
| A2#5b | 270 | 0 | 0% | 2 | 0 | 0 | 0 |
| A2#5c | 287 | 0 | 0% | 3 | 0 | 0 | 0 |
| B1#1a | 259 | 0 | 0% | 2 | 0 | 0 | 0 |
| B1#1b | 244 | 1 | 0% | 1 | 0 | 0 | 0 |
| B1#1c | 231 | 0 | 0% | 1 | 0 | 0 | 0 |
| B1#2a | 140 | 9 | 6% | 60 | 6 | 0 | 2 |
| B1#2b | 142 | 4 | 3% | 81 | 5 | 0 | 2 |
| B1#2c | 155 | 4 | 3% | 59 | 4 | 0 | 3 |
| B1#3a | 219 | 0 | 0% | 3 | 0 | 0 | 0 |
| B1#3b | 203 | 0 | 0% | 1 | 0 | 0 | 0 |
| B1#3c | 187 | 0 | 0% | 2 | 0 | 0 | 0 |
| B1#4a | 294 | 1 | 0% | 2 | 0 | 0 | 0 |
| B1#4b | 250 | 0 | 0% | 4 | 0 | 0 | 0 |
| B1#4c | 335 | 1 | 0% | 2 | 0 | 0 | 0 |
| B1#5a | 183 | 4 | 2% | 30 | 2 | 0 | 0 |
| B1#5b | 192 | 3 | 2% | 36 | 3 | 0 | 0 |
| B1#5c | 205 | 0 | 0% | 41 | 1 | 0 | 1 |
| B2#1a | 224 | 1 | 0% | 1 | 0 | 1 | 0 |
| B2#1b | 230 | 1 | 0% | 0 | 0 | 0 | 0 |
| B2#1c | 253 | 0 | 0% | 1 | 0 | 0 | 0 |
| B2#2a | 154 | 0 | 0% | 23 | 0 | 0 | 0 |
| B2#2b | 203 | 1 | 0% | 30 | 0 | 0 | 0 |
| B2#2c | 197 | 0 | 0% | 25 | 0 | 0 | 0 |
| B2#3a | 252 | 0 | 0% | 1 | 0 | 0 | 0 |
| B2#3b | 255 | 0 | 0% | 1 | 0 | 0 | 0 |
| B2#3c | 249 | 1 | 0% | 1 | 0 | 0 | 0 |
| B2#4a | 222 | 0 | 0% | 4 | 0 | 0 | 0 |
| B2#4b | 204 | 0 | 0% | 2 | 0 | 0 | 0 |
| B2#4c | 249 | 1 | 0% | 2 | 0 | 0 | 0 |
| B2#5a | 243 | 0 | 0% | 1 | 0 | 0 | 0 |
| B2#5b | 277 | 0 | 0% | 4 | 0 | 0 | 0 |
| B2#5c | 262 | 2 | 1% | 2 | 0 | 0 | 0 |
| C#2a | 195 | 24 | 12% | 1 | 1 | 0 | 0 |
| C#2b | 155 | 16 | 10% | 1 | 1 | 0 | 1 |
| C#2c | 184 | 14 | 8% | 1 | 1 | 0 | 0 |
| C#3a | 167 | 6 | 4% | 56 | 3 | 0 | 2 |
| C#3b | 158 | 7 | 4% | 51 | 1 | 0 | 1 |
| C#3c | 158 | 6 | 4% | 42 | 1 | 0 | 1 |
| C#4a | 151 | 20 | 13% | 0 | 0 | 0 | 0 |
| C#4b | 183 | 25 | 14% | 1 | 1 | 0 | 0 |
| C#4c | 161 | 17 | 11% | 0 | 0 | 0 | 0 |
| C#5a | 148 | 2 | 1% | 42 | 0 | 0 | 0 |
| C#5b | 155 | 2 | 1% | 39 | 0 | 0 | 0 |
| C#5c | 185 | 2 | 1% | 60 | 2 | 0 | 1 |
|  |  |  | **Total** | **1310** | **154** | **3** | **50** |

Images from blood smears of mice at Day 12 were studied.
